# Minimal Hepatic Encephalopathy: A Narrative Review

**DOI:** 10.7759/cureus.113978

**Published:** 2026-08-05

**Authors:** Jamir Pitton Rissardo, Fatemeh Rashidi, Hania Moharam, Ibrahim Khalil, Meryem Bahar, Masoumeh Rashidi, Basem Ahmed, Saleh Salem, Ana Leticia Fornari Caprara, Abhishek A Chouthai, Ahmed M Kedwany, Hagar H Sayed, Omesh Prathiraja, Maleesha Jayasinghe

**Affiliations:** 1 Neurology, Cooper University Hospital, Camden, USA; 2 Medicine, Nanjing Medical University, Nanjing, CHN; 3 Medicine and Surgery, Nanjing Medical University, Nanjing, CHN; 4 Neurological Surgery, Faculty of Medicine, Alexandria University, Alexandria, EGY; 5 Medical Education, Nanjing Medical University, Nanjing, CHN; 6 Dermatology, Laser Treatment, and Aesthetics, Nanjing Medical University, Nanjing, CHN; 7 Neurovascular Intervention, Nanjing Medical University, Nanjing, CHN; 8 Neurology, Universidade Federal de Santa Maria, Santa Maria, BRA; 9 Gastroenterology and Hepatology, Saint Peter’s University Hospital, New Brunswick, USA; 10 Internal Medicine, Faculty of Medicine, Assiut University, Assiut, EGY; 11 Nuclear Medicine, South Egypt Cancer Institute, Assiut University, Assiut, EGY

**Keywords:** chronic liver diseases, driving impairment, electroencephalography, hepatic encephalopathy, minimal he, minimal hepatic encephalopathy, neurocognitive testing, neuropsychological testing, psychometric tests, quality of life

## Abstract

Minimal hepatic encephalopathy (MHE) is primarily a cognitive disorder linked to chronic liver disease that often remains underdiagnosed due to the subtlety of its clinical manifestations. These symptoms are frequently overlooked or dismissed as inconsequential in clinical practice, leading to many undiagnosed cases. The standard neurological examination is usually normal in individuals with MHE. Regular and systematic screening for MHE is essential for early detection, which can improve cognitive outcomes and prevent progression to a more serious and debilitating condition known as overt hepatic encephalopathy. The pathophysiology of MHE is very complex and multifactorial, involving several interacting mechanisms. Mainly, hyperammonemia, systemic inflammation, gut-derived neurotoxins, oxidative stress, and mitochondrial dysfunction are at its root. Any of these factors may interfere with the normal functioning of neurotransmitters, giving rise to the typical subtle cognitive impairments that are hallmarks of MHE. The Animal Naming Test and EncephalApp-Stroop test are practical, rapid, and easily administered tools for screening for minimal hepatic encephalopathy in outpatient and bedside settings. Emerging diagnostic technologies, including advanced neuroimaging techniques and novel biomarkers, have also been investigated and show promise for improving the early detection and diagnosis of MHE.

## Introduction and background

Chronic liver disease (CLD) and the resulting cirrhosis lead to around 1 million deaths annually. Beyond its clinical impact, CLD significantly affects health-related quality of life (HRQL) and imposes an economic burden. While there are various causes of liver disease, the four primary contributors to the burden associated with CLD are chronic hepatitis C virus (CHC), chronic hepatitis B (CHB) virus, alcohol-related liver disease (ALD), and nonalcoholic fatty liver disease (NAFLD) [1]. Over 100 million people in the United States experience some form of liver disease. Approximately 4.5 million U.S. adults (1.8%) have received a liver disease diagnosis, but it is estimated that 80-100 million adults in the United States have fatty liver disease, with many unaware of their condition. In 2020, 51,642 adults in the United States died from liver disease [2]. Despite the availability of a highly effective vaccine and antiviral regimens for treatment and cure, the global burden of CLD continues to increase, most likely owing to the increasing rates of NAFLD and ALD [2].

CLD can manifest with nonspecific symptoms such as fatigue, anorexia, and weight loss. However, the specific presentation depends on the complications the patient has developed. The major complications of CLD include portal hypertension (leading to esophageal varices and ascites), hepatocellular insufficiency (resulting in jaundice and hepatic encephalopathy (HE)), and hepatocellular carcinoma.

HE is a significant complication associated with severe acute or chronic liver insufficiency. HE is a condition characterized by brain dysfunction resulting from liver insufficiency or portosystemic shunting [3]. It presents with a wide range of neurological or psychiatric abnormalities, spanning from subtle subclinical changes to coma. Notably, this definition of HE does not consider the underlying cause of liver disease. However, various factors associated with CLDs, including ALD, NAFLD, viral hepatitis, and primary biliary cholangitis, can independently impact brain function beyond the effects triggered by liver failure or dysfunction [4].

HE primarily manifests as alterations in personality, consciousness, cognition, and motor function. Traditionally, three types of HE have been identified based on their underlying causes: type A occurs as an essential component of acute liver failure; type B arises due to portosystemic shunts in the absence of liver dysfunction; and type C presents in patients with liver cirrhosis and portosystemic bypass. There is ongoing discussion about whether HE in patients with acute-on-chronic liver failure should be classified separately (as type D), given its distinct clinical, pathophysiological, and prognostic features [5].

**Figure 1:**
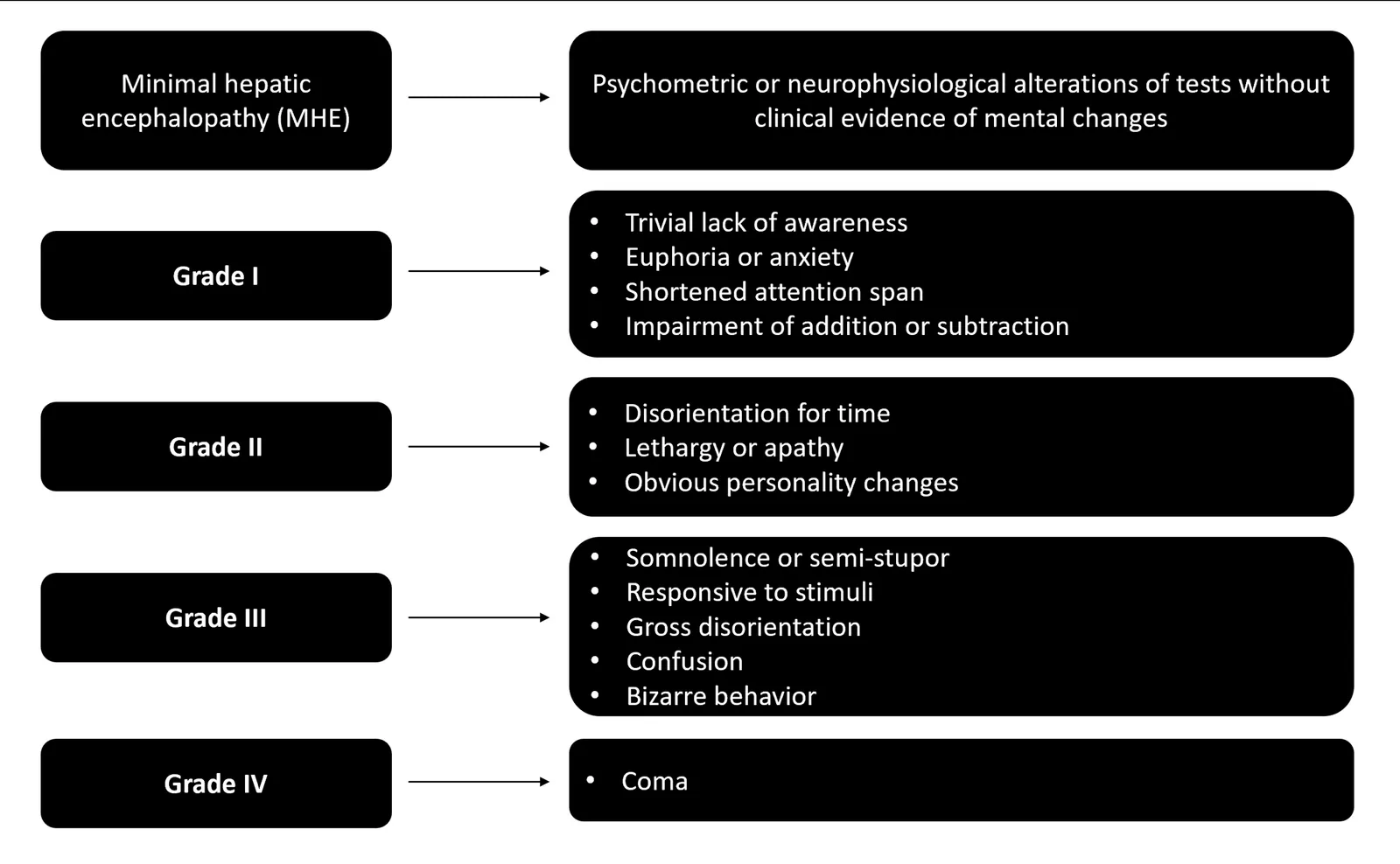
Grades of hepatic encephalopathy Original figure created by the authors using PowerPoint (Microsoft Corporation, Redmond, Washington).

The West Haven criteria (WHC) are commonly used to grade HE. This system distinguishes four clinical presentations: in Grade I, patients exhibit a lack of attention and subtle personality changes, often noticeable to their relatives; in Grade II, the most intriguing finding is disorientation to time, combined with inappropriate behavior and lethargy; in Grade III, patients are stuporous but respond to stimuli, and they are also disoriented to place and situation and may exhibit bizarre behavior; and in Grade IV, patients are in a coma. Additionally, a fifth grade was added for patients without clinical signs of HE: the so-called subclinical or minimal HE (MHE) [6] (Figure 1). The International Society for HE and Nitrogen Metabolism classifies covert HE, also known as clinically undetectable HE. According to the West Haven criteria, this category includes minimal and Grade I disease categories. Patients falling into these categories are considered to have MHE [7]. 

MHE, previously known as subclinical HE, is the earliest and mildest form of HE [8]. It needs to be better recognized and is often underdiagnosed. Patients with MHE do not display clinical evidence of cognitive changes; instead, they experience subtle alterations in psychomotor or neuropsychiatric functioning [3]. Up to 80% of patients with CLD are estimated to experience MHE [9]. While there is no definitive gold standard for diagnosing MHE, several validated testing methods have been developed to detect this neurocognitive complication. Additionally, some of these diagnostic tools can also predict the risk of progression to overt HE (OHE) [10].

While diagnosing MHE can be challenging, early detection is crucial due to its association with worse clinical outcomes. Patients with MHE face an increased risk of hospitalization and motor vehicle collisions [11]. Moreover, individuals with MHE are more likely to progress to OHE, experience impaired quality of life, and have higher overall mortality rates [12]. Therefore, this narrative review aims to provide an overview of the current literature on MHE, summarize its main features, and present current perspectives on diagnosis and management.

## Review

Pathophysiology

**Figure 2:**
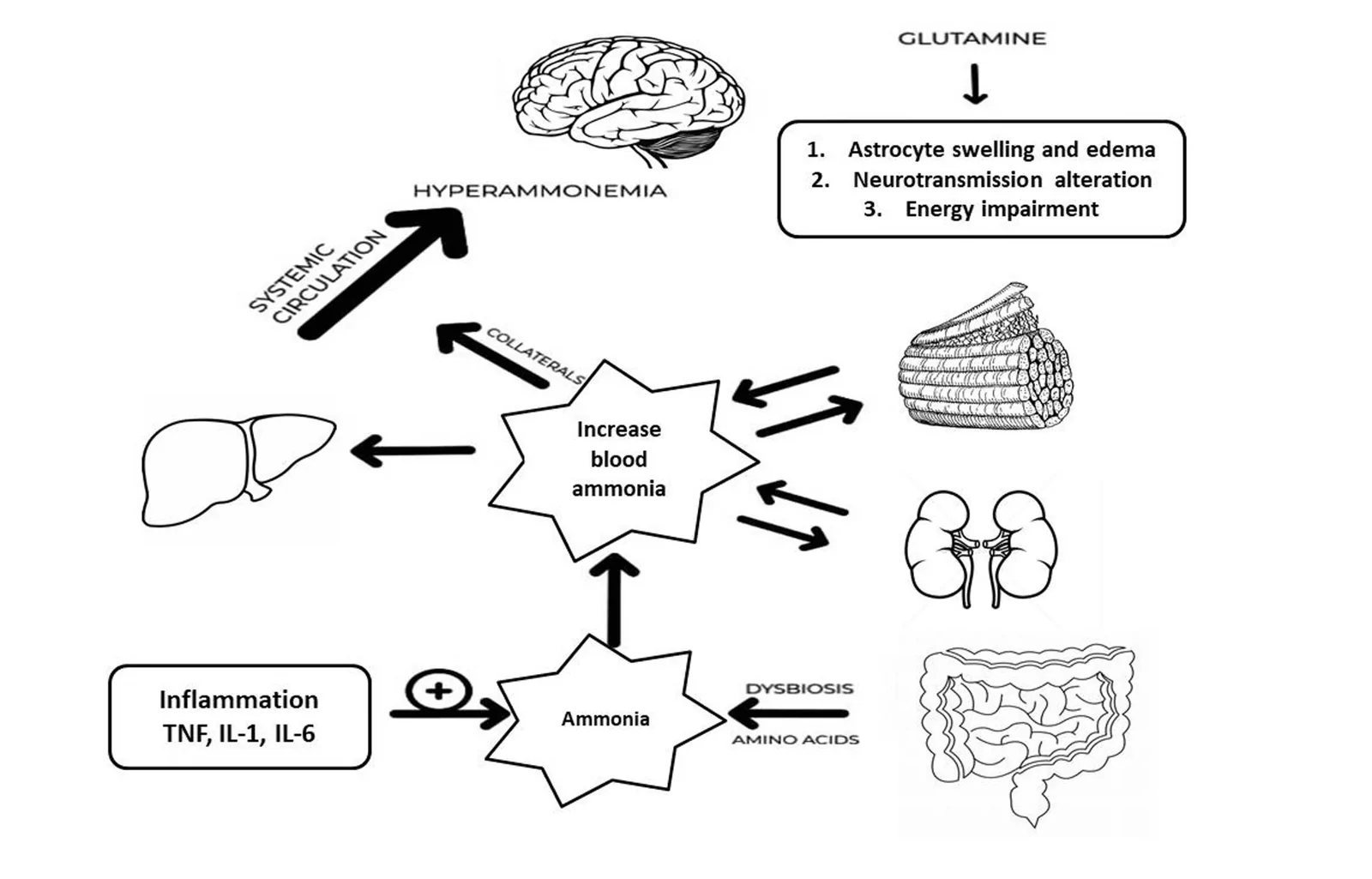
Role of ammonia in the pathophysiology of hepatic encephalopathy Original figure created by the authors using Freepik Premium (Freepik Company, S.L., Málaga, Spain).

The pathophysiology of HE is complex, as it is multifactorial, encompassing ammonia toxicity, dysregulation of central nervous system (CNS) activity, and an excess of inflammatory cytokines. Ammonia plays a crucial role in the pathophysiology of HE. Ammonia is a small nitrogenous metabolite that results from the breakdown of proteins and amino acids. While the pathophysiology of HE involves multiple factors, extensive research has centered on the accumulation of this nitrogenous waste product in the blood and brain. Although hyperammonemia is not the sole mechanism in the development of HE, it significantly impacts cellular function (Figure 2).

Role of Ammonia

Ammonia production: Ammonia is primarily produced in the small and large intestines (50%) and the kidneys (40%). In the intestine, there are two mechanisms: (1) urea from dietary protein is broken down into ammonia and carbon dioxide by urease-producing bacteria (such as *Klebsiella* and *Proteus*), mainly in the large intestine, and (2) glutamate is directly degraded into glutamate and free ammonia by glutaminase in enterocytes. This unionized ammonia can freely cross the intestinal epithelium and enter the portal circulation. The ionization of ammonia depends on the colonic luminal pH. A more acidic pH leads to protonation and trapping of ammonium (NH₄⁺) within the lumen. Additionally, nonabsorbable disaccharides, such as lactulose, can lower colonic pH, thereby reducing ammonia reabsorption [13].

While gut bacteria contribute significantly to ammonia production through protein breakdown, low-protein diets should be avoided in patients with HE. Protein malnutrition can lead to sarcopenia, which is associated with higher mortality and an increased risk of HE. Muscles play a crucial role in nitrogen metabolism for patients with cirrhosis. Additionally, increased fiber intake reduces bowel transit time and decreases ammonia absorption [14].

Renal ammoniagenesis occurs in proximal tubular cells, which generate ammonia from glutamine and produce bicarbonate as a byproduct. Renal ammonia production is influenced by acid-base status and potassium balance. In metabolic acidosis or hypokalemia, glutamine helps dispose of acid or replenish potassium, leading to increased arterial ammonia levels [15]. Intravascular hypovolemia (e.g., due to gastrointestinal bleeding or overdiuresis) enhances renal ammonia production via angiotensin II. Volume expansion (e.g., stopping diuretics) benefits patients with worsening HE [16].

Muscles also make a minor contribution by releasing ammonia through adenylic acid metabolism (part of the purine nucleotide cycle). Senescent red blood cells release glutamine, contributing to ammonia production [17].

Ammonia excretion: The primary site of ammonia breakdown occurs in the liver through the urea cycle (also known as the Krebs-Henseleit cycle). In this process, two ammonia molecules and one carbon dioxide molecule combine to form urea and water. Due to its small size, urea can easily cross membranes and return to the intestines and kidneys for excretion. Interestingly, the ammonia concentration in the portal vein is 5-10 times higher than in the systemic circulation. When liver function is compromised-either due to hepatocellular damage or shunting around the liver (such as from portal hypertension or portosystemic shunts)-ammonia accumulates in the systemic circulation [17].

The kidneys contribute to ammonia excretion by generating ammonium through parallel H1 and ammonia secretion in the collecting duct. Most urinary ammonia is produced by renal ammonia genesis. Factors like acid-base balance, potassium levels, protein intake, and glucocorticoid hormones influence the proportion of ammonia excreted in urine. Interestingly, renal venous ammonia exceeds arterial ammonia, indicating that the kidneys actually increase systemic ammonia [18]. In normal physiology, about 50% of ammonia is excreted in the urine, while the remaining 50% is returned to the systemic circulation via the renal vein. In acidosis, the kidneys excrete hydrogen ions and convert ammonia to ammonium. Conversely, alkalosis reduces renal ammonia excretion, and reduced renal perfusion (e.g., dehydration) also decreases ammonia excretion [17].

In patients with cirrhosis, skeletal muscles play a significant role in detoxifying ammonia by converting it into glutamine via glutamine synthetase (GS). Sarcopenia (muscle loss) is a risk factor for HE, so improving muscle mass is beneficial. Abnormal ratios of branched-chain amino acids (BCAAs) and aromatic amino acids in cirrhotic patients affect ammonia metabolism. BCAA supplementation has shown promise in treating HE, especially in non-responsive cases [19].

Therapeutic options such as L-ornithine L-aspartate (LOLA) enhance both ureagenesis and glutamine synthesis. L-ornithine plays a role in the urea cycle in periportal hepatocytes, while L-aspartate contributes to glutamate production via transamination in perivenous hepatocytes, skeletal muscle, and the brain. Intravenous LOLA has improved psychometric testing and postprandial venous ammonia levels in patients with persistent HE [20].

Ammonia neurotoxicity: Under normal conditions, most ammonia exists in the weakly acidic NH₄⁺ form. Interestingly, this ion has ionic radii and diffusion coefficients similar to those of potassium (K⁺). As a result, it can compete with and bind to various K⁺ channels and transporters. Ammonia can cross the blood-brain barrier by passive diffusion in its uncharged form and by active transport via ion transporters in its NH₄⁺ form [21]. Some evidence suggests that specific mammalian ammonia transporters and aquaporin channels (such as aquaporin-8) are involved in this process [22].

Astrocyte dysfunction theory: Ammonia, when detoxified by astrocytes, can induce cellular changes that alter brain function. These changes include pH shifts, alterations in membrane potential, and impaired oxidative metabolism [23]. Glutamine synthetase (GS) plays a key role in detoxifying ammonia by converting it into glutamine. However, excessive ammonia can cause astrocyte swelling and dysfunction [24]. CLD results in mild swelling, while ALF leads to significant swelling and increased intracranial pressure [25]. Additionally, hyponatremia can contribute to astrocyte swelling. Therefore, correcting electrolyte imbalances is crucial for preventing or treating HE [26].

In addition to astrocyte dysfunction, neuropathological studies have reported microglial activation [27]. This leads to a pro-inflammatory state, monocyte recruitment, and increased cytokine gene expression in the brain. PET imaging reveals heightened signal intensity in the anterior cingulate cortex, which is associated with attention control. Neuronal damage and death contribute to acquired hepatocerebral degeneration, post-shunt myelopathy, and direct cerebellar damage [28]. Liver failure can also cause neuronal death in Wernicke encephalopathy due to impaired thiamine synthesis. Mechanisms of neuronal damage include NMDA receptor-mediated excitotoxicity, lactic acidosis, oxidative stress, and proinflammatory cytokines. Despite ammonia’s role in HE, other factors play a significant role, and ammonia levels do not always correlate directly with HE severity. Neurotoxins, nutritional deficiencies, inflammation, and changes in neurotransmission are major contributors to HE [29].

Role of tumor necrosis factor-alpha: Systemic inflammation plays a crucial role in HE. Cirrhotic patients often have elevated levels of tumor necrosis factor (TNF)-alpha. Common triggers include infection or direct hepatocellular injury, leading to the release of proinflammatory cytokines like TNF-alpha, interleukin (IL)-1B, and IL-6 [30]. Gut flora translocation contributes to increased TNF-alpha levels. Additionally, proton pump inhibitors (PPIs) may exacerbate bacterial translocation. TNF-alpha affects neurotransmission, astrocyte function, and blood-brain barrier permeability, emphasizing its role in HE [31].

Role of gut microbiota: gut-liver-brain axis: Changes in gut flora contribute to HE. Reduced bile acid production in liver disease leads to pathogenic bacteria (e.g., Enterobacteriaceae) proliferation and reduced protective commensals (e.g., Lachnospiraceae) [32]. Patients with HE exhibit dysbiosis, hyperammonemia, and systemic inflammation. Specific gut microbial changes correlate with inflammation and neuronal dysfunction. Probiotics may reduce ammonia and endotoxin levels, improving HE. Fecal microbiota transplant (FMT) shows promise in treating recurrent HE [33].

Neurotransmitters and Minerals Involved in the Pathophysiology of HE

Glutamate: In HE, glutamate, the brain’s main excitatory neurotransmitter, experiences reduced transmission. Elevated ammonia levels within the CNS lead to various changes in the glutamate system [34]. These include altered expression and activity of glutamate transporters in the synaptic cleft, reduced glutamate release from nerve terminals due to decreased glutamine synthesis, prevention of action potential invasion into presynaptic terminals, and blockage of glutamate receptors [35]. These alterations contribute to the neurological symptoms observed in HE. Additionally, acute ammonia elevations lead to excessive NMDA receptor activation, resulting in significant neurotoxicity, although this effect is not seen with chronic hyperammonemia [36].

Monoamines (histamine and serotonin): The monoamine system, affected by ammonia, plays a role in HE. The activation of histaminergic systems contributes to symptoms such as disturbances in the sleep cycle and altered motor function. Studies have reported elevated histamine levels in plasma and the brain tissue, possibly due to changes in the brain’s neutral amino acid transporter system [37]. Additionally, increased concentrations of 5-hydroxyindoleacetic acid (5-HIAA), a serotonin (5-HT) metabolite, correlate with plasma ammonia levels. In patients with cirrhosis who died in hepatic comas, cerebrospinal fluid (CSF) 5-HIAA levels were elevated, suggesting increased 5-HT turnover and metabolism [38]. Mechanisms include stimulation of precursor L-tryptophan uptake, increased expression of monoamine oxidase A, and altered 5-HT storage and release from presynaptic terminals. These changes contribute to neuropsychiatric symptoms such as depression and disrupted sleep patterns [39].

Gamma-aminobutyric acid: GABA is the primary inhibitory neurotransmitter in the brain. Increased GABAergic transmission has been linked to HE [40]. Factors contributing to this include upregulation of the 18-kDa translator protein, which produces endogenous benzodiazepines (neurosteroids) that modulate GABA type A receptors. Additionally, increased peripheral benzodiazepine receptor concentrations in astrocyte mitochondria may alter energy metabolism. Clinical studies using benzodiazepine site antagonists like flumazenil have shown improvement in HE patients. The exact pathophysiology remains incompletely understood, but it involves changes in gene expression, increased brain GABA agonist activity, and endogenous modulators [41]. Allopregnanolone, a neurosteroid activating GABA type A receptors, is elevated in advanced HE. The agent GR 3027, a neurosteroid-site antagonist, shows promise in symptom improvement [42].

Manganese: Manganese (Mn) is essential for physiological functions but can become a neurotoxin in end-stage liver disease. Elevated Mn levels due to CLD lead to CNS deposition, primarily in the basal ganglia [43]. Astrocytes are particularly affected. Mn disrupts oxidative phosphorylation by sequestering in mitochondria. Impaired glutamate transport and dopamine-D2 binding result in extrapyramidal symptoms similar to Parkinson’s disease. L-Dopa treatment may improve symptoms, and liver transplantation can resolve them in some patients [44].

Risk Factors Contributing to the Development of Minimal HE

In a multicenter, cross-sectional study, 1879 patients with confirmed cirrhosis were enrolled across 40 hospitals [45]. Researchers conducted both univariate and multivariate logistic analyses to explore risk factors for MHE in cirrhotic patients. The study focused on age, duration of education, etiology, and MELD-Na score. Age was identified as an independent risk factor (p < 0.001). A longer duration of education was associated with a reduced risk (p = 0.001). Etiology (p = 0.039) and MELD-Na score (p = 0.009) were also significant risk factors. For HBV-related cirrhosis, age (p = 0.001) and duration of education (p = 0.001) influenced MHE risk. For HCV-related cirrhosis, age (p = 0.009) and creatinine concentration (p = 0.042) were risk factors. No significant risk factors were found for ALD-related cirrhosis. In alcoholic cirrhosis, platelet count (p = 0.045) was associated with MHE risk.

On the other hand, risk factors for OHE in cirrhosis include MHE, prior history of OHE, sarcopenia, hyponatremia, epilepsy, type 2 diabetes mellitus, elevated creatinine levels, elevated bilirubin levels, low albumin levels, proton pump inhibitor (PPI) use, nonselective beta-blocker use, and statin use [46,47].

Mechanisms Underlying Cognitive Dysfunction in Minimal HE

Current understanding suggests that both hyperammonemia and inflammation play significant roles in causing cognitive impairment in MHE [48]. Consequently, it is not unexpected that certain diseases, which result in similar changes in ammonia levels and inflammatory factors akin to those seen in liver cirrhosis, may also lead to mild cognitive impairment affecting performance in similar psychometric tests [49].

Felipo et al. assessed cognitive function using the PHES battery of psychometric tests in patients with varying degrees of hyperammonemia and inflammation caused by different diseases, both with and without liver failure. It was noticed that all patient groups exhibit some level of inflammation [50]. However, the severity and nature of inflammation vary across patient types, with distinct contributions from elevated IL-6 and IL-18 levels. The findings indicate that mild cognitive impairment, as evaluated using the PHES battery, necessitates the simultaneous presence of moderate hyperammonemia and inflammation. While it remains possible that hyperammonemia or inflammation alone may affect other cognitive functions, impairment, specifically in the cognitive domains assessed by the PHES battery, appears to require a combination of both factors [51].

In rat models of MHE, impaired cognition is induced by reduced synthesis of cyclase guanylyl monophosphate (cGMP) through various mechanisms. This cognitive impairment is evident in reduced learning ability observed during a Y-maze task. Neuroinflammation, characterized by increased extracellular GABA and hyperammonemia that activate N-methyl-D-aspartate receptors, ultimately leads to decreased neuronal NO synthase activity, resulting in reduced cGMP synthesis [52].

Differences in Brain Activity Between Averted HE and Minimal HE

In addition to the common alterations in brain activity observed in patients with OHE and MHE, quantitative comparison analysis revealed that OHE patients exhibit reduced brain activity in regions associated with the default mode network (DMN) compared with MHE patients [53]. The DMN plays a role in various cognitive functions, including memory, visual and auditory attention, motor activity, and language processing. Specifically, the precuneus, a key component of the DMN, is closely linked to visuospatial information integration, episodic memory retrieval, and self-processing operations.

Consistent with these findings, a resting-state functional MRI (rs-fMRI) study demonstrated decreased functional connectivity within the DMN in patients with prior OHE compared to those with current MHE. These results suggest that resting-state brain activity in the DMN could serve as a noninvasive, sensitive neuroimaging biomarker for detecting progression from MHE to OHE. Furthermore, OHE patients exhibited increased brain activity in specific regions, including the left olfactory cortex, left cerebellum (crus I), right superior temporal gyrus (STG), and right putamen, when compared to MHE patients. Such increased activity is often interpreted as a compensatory mechanism resulting from neural reorganization. Thus, the heightened brain activity in these regions among OHE patients may play a compensatory role as the severity of HE worsens. These findings suggest that rs-fMRI could be a valuable imaging technique for assessing HE severity in clinical practice [54].

Definition 

Modern diagnostic methods enable the detection of functional abnormalities even before symptoms manifest. Identifying impairments early can help prevent or delay disease progression. This approach is essential for recognizing and modifying risk factors, such as hypercholesterolemia or arterial hypertension [55]. Neurologists, recognizing the significance of early Alzheimer’s disease diagnosis, coined the term “mild cognitive impairment” to identify patients in the initial stages of the disease, candidates who may benefit from available treatments. Similarly, early identification of patients with HE can improve their quality of life and prognosis.

The concept of “subclinical HE” was introduced to identify patients exhibiting subtle manifestations of HE that are challenging to recognize. This term underscores the importance of using complementary tests to diagnose brain dysfunction that might not be detectable through standard clinical examination [56].

Other researchers have preferred terms like “latent” or “early” to emphasize the potential progression to OHE [57,58]. However, once the impact on patients’ quality of life became evident, the term “subclinical” faced criticism. To prevent medical misunderstandings, alternative terms were suggested to avoid the implication that the condition is insignificant [59]. The current consensus is to use the term “minimal hepatic encephalopathy” (MHE), as proposed by the Working Party commissioned by the XI World Congress of Gastroenterology [60]. The American Association for the Study of Liver Diseases (AASLD) defines MHE as the presence of test-dependent or clinical signs of brain dysfunction in cirrhotic patients who are not disoriented or displaying asterixis [3].

Clinical Manifestations

The International Society for HE and Nitrogen Metabolism (ISHEN) consensus delineated the symptoms and their grading into covert (MHE and grade I) and OHE (OHE; grades II-IV) in September 2014. Motor manifestations of stage I HE begin with impaired handwriting and incoordination. Stage II HE is characterized by asterixis. As encephalopathy progresses, flapping tremors weaken in stage III and disappear in stage IV. Later stages involve hyporeflexia, ataxia, hyperreflexia, clonus, and rigidity and may culminate in opisthotonus and coma [3].

MHE is often underrecognized, significantly impacting patients’ quality of life and their families. It is highly prevalent among cirrhotic patients, affecting up to 80% of this population. Despite normal clinical examination findings, patients with MHE exhibit abnormal psychometric test results. These subtle symptoms necessitate specialized neuropsychiatric diagnostic testing [61]. MHE primarily affects executive functioning, leading to deficits in vigilance, response inhibition, working memory, and orientation [62]. Importantly, MHE can impair daily functioning, including safe automobile operation [63]. The ISHEN consensus defines the onset of OHE as the appearance of disorientation or asterixis [64]. As awareness of MHE grows, early treatment may significantly enhance the quality of life for cirrhotic patients.

Clinical Significance

Effect of minimal HE on quality of life: QOL encompasses physical, cognitive, emotional, and psychosocial well-being. It is assessed using various scales, such as the Nottingham Health Profile (NHP), the Sickness Impact Profile (SIP), and disease-specific questionnaires [65,66].

MHE negatively impacts HRQOL. Complex tasks requiring attention, information processing, and psychomotor skills are affected. Basic self-care functions remain preserved [67]. Patients with MHE show impaired scores across SIP scales, affecting social interaction, alertness, emotional behavior, sleep, work, and recreation [68].

Minor symptoms (e.g., pruritus, muscle cramps) impact HRQOL. Depression, anxiety, and malnutrition worsen HRQOL [69]. Lactulose, rifaximin, and probiotics can reverse MHE and improve HRQOL. Driving performance also improves after MHE reversal with rifaximin therapy [70].

Sleep and HRQOL: Sleep disturbances are common in patients with cirrhosis, affecting 26-70% of cases. Patients with MHE experience more sleep disturbances than those without MHE. Homeostatic and circadian processes regulate sleep. MHE disrupts these processes. Unsatisfactory night sleep, delayed onset, and multiple awakenings lead to reduced sleep time and daytime sleepiness [71]. Excessive daytime sleepiness correlates with ammonia levels and increases HE-related hospitalization risk. Sleep disturbances can impair HRQOL in patients with MHE [72].

Memory and learning difficulties in MHE: MHE involves subcortical dysfunction, including slowed mental processing, attention disturbances, executive disabilities, psychomotor slowing, and memory issues. MHE primarily affects short-term memory due to attention deficits, leading to encoding defects and learning impairments [73]. FDG PET studies reveal decreased glucose metabolism in the parieto-occipital region (related to visual perception) and preserved metabolism in the temporomesial area (associated with memory) [74]. Learning impairment in MHE may result from hyperammonemia, which can affect cGMP levels. Pharmacological interventions may help [75].

Driving and navigational skills in MHE: MHE patients show impaired driving performance in real-world and simulator tests. The categories most affected include car handling, maneuvering, adaptation, and cautiousness [76]. The increased risk of accidents is attributed to cognitive decline in MHE patients. Difficulties in following maps and higher self-reported traffic violations are observed. Fatigue worsens driving performance over time. Navigation relies on working memory, attention, response inhibition, visuomotor coordination, and executive control [77]. MHE patients have navigation difficulties, leading to illegal turns and accidents. Rifaximin improves driving performance in MHE patients, correlating with cognitive improvement [11].

Falls in MHE

MHE is associated with an elevated risk of falls, which can impact patients’ quality of life. While only 12% of patients without MHE experience falls, nearly 40% of those with MHE suffer falls that often require hospitalization [78]. The risk is further heightened for patients taking psychoactive drugs. Impaired attention, visuomotor coordination, and slowed reaction time increase the fall risk in MHE patients [79]. Additionally, the higher incidence of falls in these patients can lead to osteoporosis-related fractures, surgeries, and associated morbidity, ultimately affecting both patients and their families [80].

Employment and socioeconomic burden of MHE: Patients with MHE experience reduced work performance due to psychomotor slowing [81]. Nearly half are unemployed, compared to only 15% of patients without MHE. Blue-collar workers are particularly affected, with 60% unfit for work, while verbal intelligence remains preserved [82].

Natural history and survival in MHE: Patients with MHE can experience improvement, stability, or deterioration over the long term. The frequency of MHE increases with the severity of liver dysfunction. MHE predicts the development of OHE and adversely affects survival [83]. Studies have shown that both abnormal psychometric scores for HE and the Child-Turcotte-Pugh (CTP) score, a widely used clinical scoring system for assessing the severity and prognosis of CLD, independently correlate with survival. Additionally, covert HE is associated with worsened survival, increased hospitalization risk, and development of overt HE [84]. Additionally, covert HE is associated with worsened survival, increased hospitalization risk, and overt HE development [84].

Diagnosis and available tests

**Figure 3:**
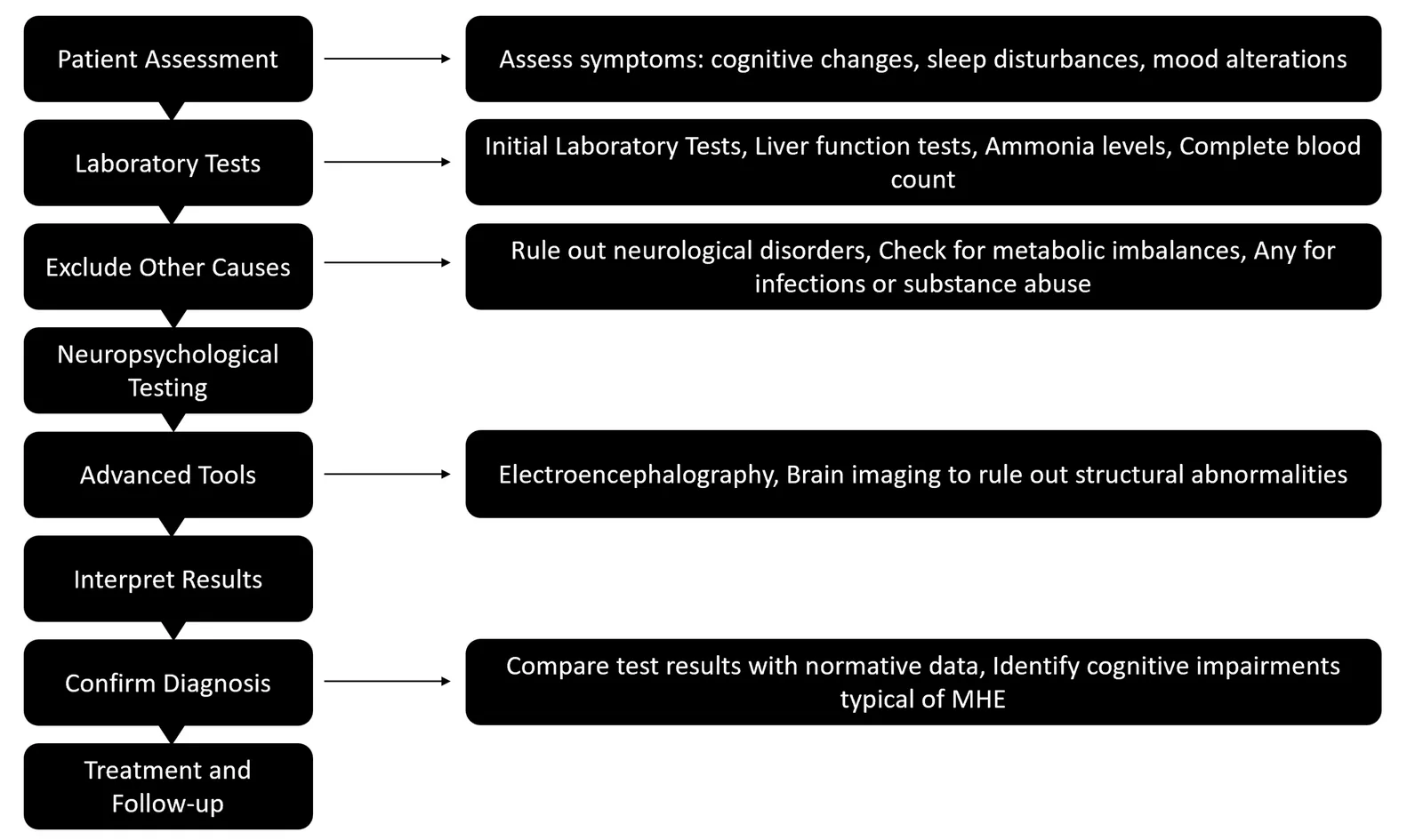
The algorithm for arriving at the diagnostically appropriate diagnosis of minimal hepatic encephalopathy Original figure created by the authors using PowerPoint (Microsoft Corporation, Redmond, Washington).

MHE is the mildest clinical manifestation of HE. It can impact as many as 80% of individuals with liver cirrhosis, varying depending on the specific population examined and the diagnostic method employed [85]. Thus, every patient who is at risk should undergo testing for this condition. This is because MHE is a significant health issue and, even in its mildest form, it is linked to carrier burden, poor prognosis, increased risk of experiencing episodes of OHE, inability to drive, sleep disorders, falls, and ultimately, an impairment of the quality of life [86,87]. The most effective method for identifying MHE is still an ongoing issue. None of the offered strategies adequately addresses the intricate and diverse nature of cognitive impairment in MHE (Figure 3).

Additionally, appropriate norms are often necessary, and MHE is still often unrecognized or underdiagnosed by most clinicians [88]. The diagnosis of MHE may be frequently missed due to various factors, including the fact that MHE is challenging to diagnose solely on objective neurological examination, which requires specific neuropsychological and neurophysiological studies, and that cognitive impairment can affect overall performance and psychomotor skills [9]. In contrast, verbal skills typically remain intact [85]. Certain tests can be costly and necessitate highly skilled personnel and specialized testing equipment [83]. The absence of diagnostic criteria and age- and education-adjusted normal distribution values is notable [85]. There is no singularly ideal approach to evaluating the existence of MHE, as none of the proposed tests comprehensively addresses all aspects of HE; each method investigates distinct brain functions [9].

Psychometric Testing

Psychometric testing is a cornerstone of diagnosing MHE, providing valuable insights into the often-subtle cognitive impairments that accompany this condition. Sharma et al. reported that these tests assess a range of psychological functions frequently impacted by MHE, including attention, memory, and visuospatial abilities. Psychometric Hepatic Encephalopathy Score (PHES), specifically designed and validated across numerous populations and clinical scenarios to detect and stage HE [89]. For a broader neuropsychological evaluation, the Repeatable Battery for the Assessment of Neuropsychological Status (RBANS), also described in the table, can identify cognitive deficits, including those characteristic of MHE [90]. Even simpler tests like the ANT, measuring verbal fluency, a function often impaired in MHE, can be helpful [91]. Factors such as age, education level, and previous test experience can influence results, making it essential to interpret findings carefully and within the context of each patient's overall clinical picture.

Furthermore, the field is continuously evolving, with ongoing research refining existing tests and exploring innovative digital assessments, such as EncephalApp [92] and QuickStroop [93], for their potential advantages in accessibility and ease of administration. Ultimately, a comprehensive approach to diagnosing MHE often involves integrating findings from various psychometric tests, clinical observations, and laboratory markers to gain a holistic understanding of each patient's condition [94]. A summary of diagnostic methods for detecting MHE is presented in Table 1.

**Table 1:** Diagnostic methods for the detection of MHE ANT: Animal Naming Test, CFF: critical flicker frequency testing, CHE: covert hepatic encephalopathy, CRT: Continuous Response Time test, EEG: electroencephalography, HRQOL: health-related quality of life, ICT: Inhibitory Control Test, MHE: minimal hepatic encephalopathy, PHES: Psychometric Hepatic Encephalopathy Score, RBANS: Repeatable Battery for the Assessment of Neuropsychological Status, SIP: Sickness Impact Profile, 3-NT: 3-nitrotyrosine.

Method	Advantages	Disadvantages	References
Psychometric testing			
PHES	-Validation across many populations and a wide range of therapeutic instances -Assists in diagnosing MHE and predicting the developing OHE. -High sensitivity and specificity when diagnosing MHE	-Insufficient localization -Age and education levels greatly affect scores, requiring adjustment and complicating interpretation -Repeated use may provide practice effects when patients do better owing to familiarity with the tests rather than cognitive progress	Amodio et al. (2008) [95], Dhiman et al. (2010) [89], Duarte-Rojo et al. (2011) [96], Seo et al. (2012) [97], Li et al. (2013) [98]
RBANS	-Time limitation to be performed in the clinical setting (30 minutes) -Efficacy in evaluating cognitive deficiencies in a variety of conditions -Initial diagnosis and monitoring of cognitive changes over time	-Certain factor configurations may be more suitable for specific populations -Demographic characteristics can impact performance -May not evaluate some specific cognitive areas, such as executive function	Duff et al. (2015) [90], Schmitt et al. (2010) [99], Garcia et al. (2008) [100], McKay et al. (2008) [101]
ANT	-Convenient for regular screenings -Do not require equipment or considerable training -Correlation with other diagnostic instruments, such as the PHES and EEG	-Demographic characteristics can impact performance -Restrictive data on cognitive function requiring more extensive neuropsychological assessments -Dependence on verbal fluency might create bias, especially for those with speech or language difficulties, who may not adequately represent their cognitive capabilities	Campagna et al. (2017) [91], Labenz et al. (2019) [102], Agarwal et al. (2020) [103], Qu et al. (2021) [104], Gairing et al. (2023) [105]
CRT	-Treatment-response evaluation -Assess the consistency of reaction times -Unaffected by age and gender	-Less stable than CFF -There was no correlation between CRT and laboratory tests -Long duration, requiring around 10 minutes to be performed	Lauridsen et al. (2015) [10], Lauridsen et al. (2017) [106]
ICT	-Notable level of sensitivity (88-90%) and specificity (up to 90%) in the diagnosis of MHE -Goog prediction for the onset of OHE -Strong test-retest reliability	-Weak correlation between ICT results and the severity of liver disease (Child-Turcotte-Pugh or MELD scores) -ICT may have lower diagnosis accuracy than alternative approaches like the PHES	Taneja et al. (2012) [107], Goldbecker et al. (2013) [108], Gupta et al. (2015) [109]
EncephalApp (Stroop test)	-Accessible in clinical and non-clinical environments -Strong test-retest reliability -Weakly affected by demographic factors -Correlation of scores with sleep disruptions in patients with cirrhosis	-Learning effect with repeated testing -Affected by age, education level, and smartphone experience -The ideal threshold values for detecting MHE using EncephalApp exhibit significant variation among different studies	Bajaj et al. (2015) [92], Luo et al. (2020) [110], Cunha-Silva et al. (2022) [111], Lupescu et al. (2023) [112]
QuickStroop	-High accuracy and specificity to detect MHE -Can be performed within 40 seconds, potentially enhancing CHE testing and treatment	-Disadvantages are similar to the EncephalApp	Acharya et al. (2023) [93]
SIP-CHE	-High level of sensitivity (80%) and specificity (79%) in identifying MHE -Does not require specific equipment or substantial training -Useful for monitoring the progress of HRQOL and cognitive function	-Clinicometric features verified in specific populations, but their use in many cultural contexts is limited -Primarily a screening tool and does not thoroughly evaluate cognitive function (additional tests are usually necessary)	Nabi et al. (2014) [113], Maharshi et al. (2016) [114], Lauridsen et al. (2020) [115]
SCAN test	-Detects varying levels of hepatic encephalopathy by monitoring reaction times corresponding to the condition’s seriousness -High sensitivity and specificity in differentiating between different HE degrees -Impartial assessments of response times, minimizing the subjectivity	-Influenced by the patient's cooperation level and cognitive condition -The absence of established standards for the administration and interpretation in various healthcare settings leads to variations and inconsistencies in the diagnosis of MHE	Montagnese et al. (2012) [94], Luo et al. (2019) [116]
Electrophysiologic tests			
EEG monitoring	-Objective data, quantifiable parameters connected with mental state -Minor alterations in brain activity can be identified that may not be evident only by clinical assessment -EEG can be performed in various clinical environments, including those with minimal resources	-Results interpretation can be subjective (high interrater variability) -The association between EEG alterations and clinical and psychometric findings regarding MHE can sometimes be variable -Standardized signal amplification, filtration, and spectral analysis techniques are necessary for precise EEG analysis	McLaughlin et al. (1989) [117], Amodio et al. (1999) [118], Amodio et al. (2006) [119], Pascoli et al. (2006) [120], Schiff et al. (2016) [121]
Evoked potentials	-Minimally invasive, uncomplicated, and can be readily conducted, even in intensive care units -Useful for patients with different levels of consciousness because they do not rely much on patient cooperation -Specific patterns have a strong correlation with cognitive deficits and psychometric test outcomes	-Advanced equipment and specialized knowledge needed -Standardized methods for recording and interpretation are necessary	Amodio et al. (2005) [122], Kullmann et al. (1995) [123], Zeneroli et al. (1984) [124]
CFF	-High level of sensitivity (96%) and specificity (77%) in diagnosing MHE -Unaffected by the patient's age or literacy level -Predict the risk of progression to OHE	-Patient's state and the severity of liver disease can impact outcomes -There is usually no significant correlation between CFF and other psychometric tests	Sharma et al. (2007) [125], Sharma et al. (2010) [126], Lauridsen et al. (2011) [127], Barone et al. (2018) [128], Romero-Gomez et al. (2007) [83]
Laboratory tests			
Biomarkers	-Ammonia is reliable for evaluating the occurrence of HE -Unbiased biomarker that minimizes the subjective inconsistencies in psychometric assessments and identifies the risk of OHE -There is a correlation between ammonia levels and cognitive impairment in MHE	-Ammonia levels alone have limited clinical value in identifying MHE, and a comprehensive diagnostic strategy is recommended.	Direkze et al. (2015) [129], Suzuki et al. (2016) [130], Fiati et al. (2019) [131]
	-3-NT is a reliable biomarker with high sensitivity (90%) and specificity (93.33%) -Superior diagnostic accuracy of 3-NT compared to other markers like plasma ammonia	-Specialized technology and experience are necessary for detecting 3-NT -It is advisable to utilize this tool alongside other diagnostic instruments to improve precision.	Montoliu et al. (2011) [132], Salman et al. (2021) [133]

**Figure 4:**
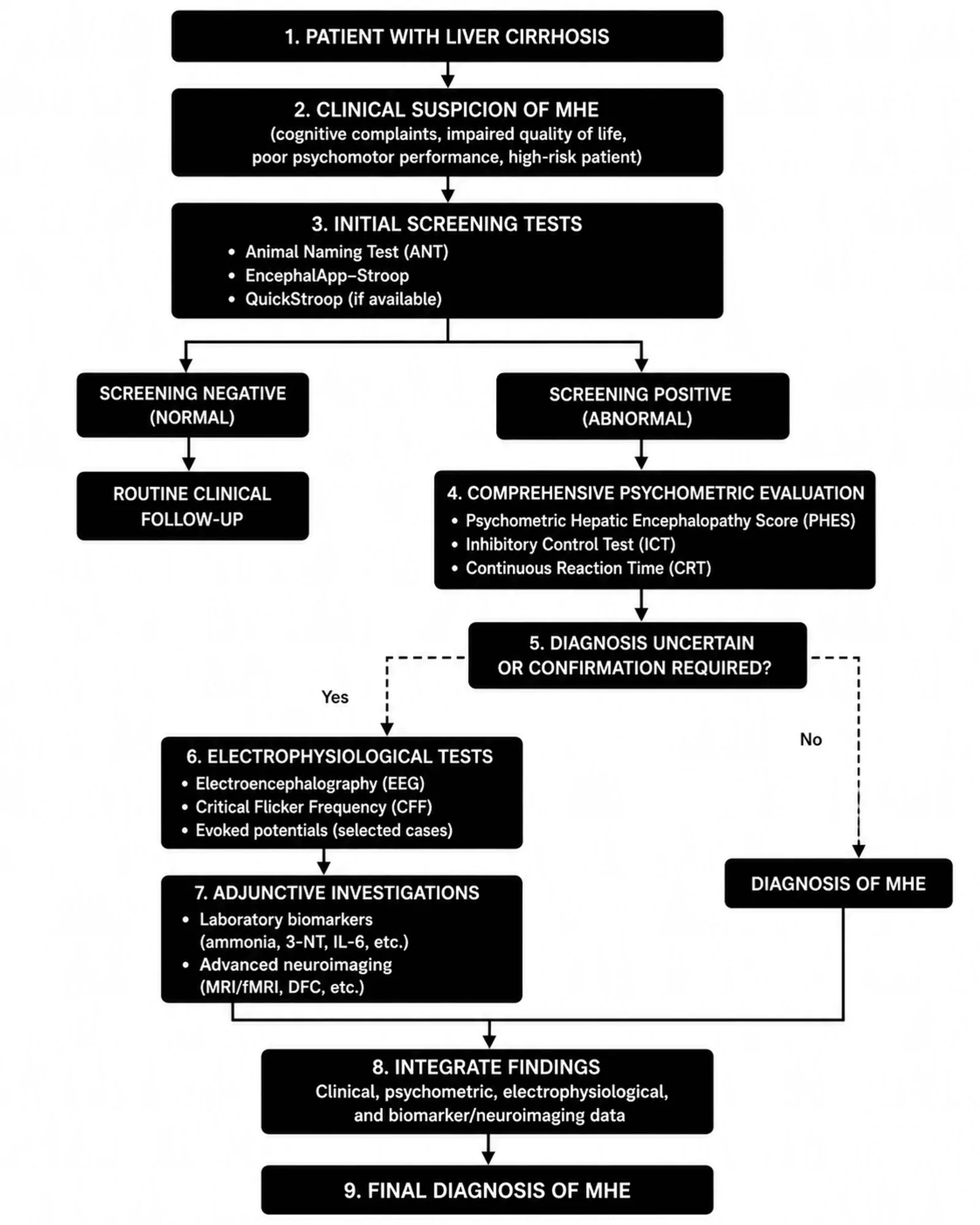
Proposed clinical algorithm for the diagnosis of minimal hepatic encephalopathy Original figure created by the authors using PowerPoint (Microsoft Corporation, Redmond, Washington).

While Table 1 summarizes the advantages and limitations of the currently available diagnostic modalities for MHE, selecting the most appropriate test in routine clinical practice can be challenging. A stepwise diagnostic approach may facilitate clinical decision-making by integrating screening, confirmatory psychometric testing, and adjunctive investigations. As illustrated in Figure 4, rapid screening tools such as the ANT and EncephalApp-Stroop may be used for initial evaluation in outpatient or bedside settings. Patients with abnormal screening results should undergo a comprehensive psychometric assessment, preferably using the PHES. Electrophysiological tests, biomarkers, and advanced neuroimaging may serve as complementary tools when the diagnosis remains uncertain or additional confirmation is required.

Psychometric Hepatic Encephalopathy Score (PHES): The PHES, a set of psychometric test techniques, is currently recognized as the worldwide preferred and most reliable method for diagnosing MHE [96]. PHES was specifically designed for individuals with MHE and has been confirmed to be reliable and valid for MHE diagnosis. The PHES consists of five tests: number connection test A, number connection test B, serial dotting test, line tracing test, and digit symbol test. The PHES evaluates various cognitive functions, including motor speed, accuracy, concentration, attention, visual perception, visual-spatial orientation, visual construction, and memory [6].

A cohort of Thai adults with cirrhosis assessed the efficacy of PHES in diagnosing MHE [134]. The authors described that a PHES threshold of ≤−5 points suggests MHE. MHE was detected in 26.6% of cirrhotic patients who did not exhibit OHE. However, some authors reported that test findings with a score (≤-2SD) were considered abnormal [6,95]. Furthermore, validation studies have been conducted in Italy [95], Spain [83], Germany [6], Korea [97], China [98], and India [89]. Standardizing these tests using a large control group must account for variations in mathematics, literacy, and language skills that may affect test outcomes [94]. The test findings are additionally impacted by factors such as age, gender, level of education, alcohol consumption, and visual abilities of the individual being tested [135]. While adjusting the findings based on age and education is possible, managing the impact of other variables is challenging. Another issue arises from the diminishing sensitivity of these tests due to many repetitions [136].

Repeatable battery for the assessment of neuropsychological status (RBANS): The RBANS has been confirmed by a working committee established by the International Society for HE and Nitrogen Metabolism (ISHEN) to evaluate the existing evidence on the neuropsychological evaluation of HE, and it is recommended for diagnosis [135]. ISHEN suggests conducting the RBANS test together with the PHES test. The RBANS assesses verbal and visual anterograde memory, working memory, cognitive processing speed, language, and visuospatial function [137]. It specifically evaluates cognitive skills that are not impacted by HE but are often affected [6]. The test battery was developed to evaluate dementia and identify cognitive impairment in other conditions. Thus far, the utilization of this method for identifying MHE has been limited [138]. The RBANS test has a low sensitivity of just 38% in accurately detecting OHE. Therefore, it is not recommended to utilize RBANS to diagnose MHE [108].

Animal Naming Test (ANT): Campagna et al. confirmed the effectiveness of the ANT in assessing cognitive impairment in individuals diagnosed with cirrhosis [91]. This highly straightforward test requires the patient to enumerate as many species of animals as they can within one minute. Furthermore, a study revealed that the ANT is an effective, uncomplicated, and flexible instrument for assessing cognitive impairment. While the data does not directly establish a connection between the ANT and HE, it does indicate that the ANT identifies a group of individuals with low health status and higher rates of frailty and impairment. This suggests that further investigation is needed to understand the underlying causes of these conditions [139].

Continuous Response Time (CRT) test: This test evaluates motor response time by instructing the patient to press a button upon hearing auditory stimuli through headphones. The CRT index is the most crucial test result as it quantifies the stability of reaction times. The test result can distinguish between organic and metabolic brain dysfunction. The test is unaffected by the patient's age or gender and does not affect learning or fatigue [140].

Inhibitory Control Test (ICT): ICT can be easily and quickly used to diagnose MHE in a clinical setting in just a few minutes [87]. The effectiveness of ICT in diagnosing MHE has been confirmed by multiple studies [141]. There was no disparity in ICT efficiency between patients with alcoholic cirrhosis and people with nonalcoholic cirrhosis [142]. These findings suggest that the various causes of liver cirrhosis do not influence the use of ICT for MHE diagnosis [143]. The findings of ICT are also not influenced by age in both cirrhotic patients and controls [144].

Moreover, the findings of ICT show improvement following lactulose therapy and deterioration after a transjugular intrahepatic portosystemic shunt. This suggests that ICT can be utilized to assess the psychometric changes caused by these treatments [143]. In clinical settings, the ICT is widely recognized as a reliable diagnostic tool for MHE. Consequently, it enables clinicians to accurately identify cirrhotic patients at heightened risk of MHE. These patients can then be recommended to undertake other neurophysiological and psychometric tests for further evaluation [109].

EncephalApp (Stroop App) and QuickStroop: They are available as mobile applications. The application has been accessible since 2013 [145]. Presently available for both iOS and Android devices. The program utilizes the Stroop effect to evaluate psychomotor speed and cognitive awareness. The objective for the patient is to determine the colors of the stimulus displayed on the screen. The comprehensive assessment comprises three steps. Initially, the patient selects the color in which a randomly chosen word is presented on the screen. In the second task, the patient must identify the color of the word that corresponds to its color definition, as indicated by its name. During the third phase, there is a discrepancy between the name of the color and the actual color of the word. The test result is promptly accessible upon completion [145]. A multicenter study comparing it to the PHES and ICT showed a high level of accuracy in detecting the condition and is effective in evaluating the likelihood of developing OHE with a favorable sensitivity (70-80%) for screening minimal MHE [146].

The Quick Stroop test is a version of the EncephalApp Stroop test and is a reliable substitute for the full EncephalApp Stroop and PHES. It can accurately and specifically detect cognitive impairment associated with rapid cognitive health evaluation in real-world scenarios [93]. Furthermore, a study by Kanagalingam et al. suggested that the QuickStroop test, which can be completed in about a minute and utilizes the early runs of the EncephalApp Stroop's "Off" stage, is a dependable method for diagnosing covert or MHE. CHE was linked to elevated odds of developing OHE and necessitating hospitalizations [147].

SCAN test: The test is a computerized assessment that evaluates the patient's ability to accurately and quickly complete a task involving the recognition of digits. The task's difficulty increases as the test progresses [94,148]. The process involves randomly presenting a sequence of 72 ordered pairs of numbers on a computer screen for three seconds. Patients are directed to press the corresponding number on a keyboard if they recognize a familiar digit in the series of digits displayed. The study records the average reaction times and the proportion of errors and then evaluates the results by considering the reaction times in relation to the number of errors [149].

Electrophysiologic Tests

Electroencephalogram monitoring: The EEG can serve as a valuable tool in the differential diagnosis of disoriented or comatose persons with liver insufficiency/shunt, particularly when the diagnosis of HE is uncertain. It can help guide the diagnosis toward alternative illnesses [150]. HE is defined by changes in the oscillatory characteristics of neural networks in the brain [151]. The EEG readily detects a broad range of irregularities, which have been conclusively linked to behavioral changes in HE. The EEG consists of two primary components: repetitive background activity and transients. Patients' HE sees a gradual decrease in the fundamental rhythmic activity of their EEG.

Furthermore, the waking EEG's responsiveness to opening the eyes diminishes and ultimately ceases. Transient phenomena, specifically called 'triphasic waves,' can be detected in the EEG of patients with moderate/severe HE. However, it is worth noting that these waves are not exclusive to this particular metabolic disorder [152]. It detects alterations in brain activity, even in uncooperative patients [9].

In individuals with MHE, quantitative electroencephalography (q-EEG) reveals an elevated proportion of theta wave activity and a reduced mean dominant frequency (MDF) in the posterior derivations. These alterations are associated with indicators of liver dysfunction and can predict the development of HE and death related to liver disease [153]. An EEG study conducted during sleep can benefit cirrhotic patients by detecting variations in MDF that early indicate brain dysfunction in individuals with MHE [118]. During this scenario, q-EEG reveals changes in the slow oscillatory activity, characterized by an elevated frequency of the prominent delta rhythm [85]. At first, the EEG does not show overall slowing but simply periods of theta activity. Quantitative EEG demonstrates no decrease in the average dominant frequency (MDF) [154]. However, it shows an increase in theta activity, specifically in the posterior scalp regions [155]. A study discussed an early EEG marker (alpha rhythm). The paper presents a new biophysical model, MHE-AWD-NCM, that includes communication dynamics between the cortical neuron population (CNP) and the astrocyte population (AP). This model aims to understand the role of disrupted communication between astrocytes and neurons in the development of alpha wave disturbances (AWD) in MHE [156].

Evoked potentials: Electrical signals can be created by adequately stimulating excitable tissues using light (visual evoked potentials (VEPs)), acoustic signals (brainstem auditory evoked potentials (AEPs)), or electrical stimulation of somatosensory nerves [123]. The latency of VEPs is determined by the passage of signals from the retina to the visual brain through several connections [157]. VEPs can be affected by several factors, including optic nerve diseases, demyelinating processes, disorders of subcortical or cortical neurons in the cerebral hemispheres, metabolic abnormalities, and the use of psychoactive medications [158]. The latency of the P100 wave of pattern visual evoked potentials (pVEPs) has been utilized to monitor MHE [159]. VEPs measure the time delay between visual input and the corresponding brain activity; however, the outcomes can vary. The variability in the assessment of subclinical HE may be attributed to the use of an N3 rather than the commonly used P100 component in routine neurological examinations. Furthermore, the definition of subclinical HE varies across studies, making it difficult to compare results. Consequently, using VEPs in patients with MHE seems to have limited diagnostic significance [160].

The significant incidence (about 40%) of flash visual evoked potential abnormalities in cirrhotic patients with normal EEG and psychometric performance indicates that this approach is highly sensitive in diagnosing HE [124,161]. Brainstem AEPs (BAEPs) are generated by delivering rapid sequences of monaural auditory stimuli, typically consisting of 1000 to 2000 clicks. Stimulation of several regions of the brainstem subsequently accompanies activation of the auditory nerve. Healthy individuals can have a total of seven detectable positive and negative waves. Patients in HE stages 0-I do not exhibit any notable elongation of BAEP-peaks I-V or the interpeak delay I-V. BAEPs exhibit erratic responses during HE testing [123].

Critical flicker frequency (CFF) testing: CFF is a widely recognized neurophysiological method that assesses the CNS's capacity to perceive rapid changes in light intensity. This measurement is directly impacted by cortical activity [162]. It involves presenting light pulses at progressively lower frequencies (starting at 60 Hz) and requiring the patient to touch a button as soon as the perception of steady light transitions to flickering light. Following a training period, the test is conducted eight times, and the average of these trials is computed as CFF, which serves as a metric of visual temporal resolution. The threshold value is between 38 and 39 Hz, and the duration is approximately 10 minutes [125]. CFF can identify a wide range of neuropsychological disorders, including issues with visual signal processing (retinal gliopathy) as well as cognitive functioning.

Furthermore, PHES investigates the interplay between visual perception, construction, visual/spatial orientation, motor speed, accuracy, focus, and attention. Therefore, the two ways could be mutually beneficial [163]. A discrepancy has been reported between neurophysiological research, which primarily focuses on externally induced brain responses, and psychometric testing [164].

Biomarkers

Ammonia is a serum biomarker that 60-95% of clinicians in their medical practice use to diagnose HE [165]. However, if a patient with OHE has a normal blood ammonia level, this should raise suspicion of a different diagnosis [136].

Hyperammonemia can cause cell death by activating N-methyl-D-aspartic acid (NMDA) receptors excessively through glutamate, as well as increasing the activity of GABAergic receptors, which can be temporarily reversed with flumazenil [166]. A research team in Valencia studied the harmful oxidant 3-nitro-tyrosine as a biomarker. The reason for this choice was the well-established fact that hyperammonemia can cause oxidative stress. Indeed, 3-nitro-tyrosine has been identified as a toxic substance that has a role in the development of HE. Their preliminary study demonstrated encouraging findings, indicating that 3-nitro-tyrosine levels above 14 nM exhibited about 90% sensitivity, specificity, positive predictive value, and negative predictive value for MHE [132]. Another study was conducted in Egypt between 2016 and 2018. The quantification of 3-nitro-tyrosine in serum is an easy and efficient process that does not require a significant amount of time, and this would enable the expansion of the diagnostic of MHE to a wide range of clinical environments, facilitating the identification of patients with MHE. 3-nitro-tyrosine serum levels serve as a reliable indicator for the existence of MHE in individuals with liver cirrhosis. These levels exhibit a high sensitivity of 90% and a specificity of 93.33%. Additionally, the positive and negative predictive values at a cutoff of 14 ng are 93.1% and 90.3%, respectively [167].

Inflammation is another factor contributing to the development of HE. Indeed, chemokines, cytokines, and ammonia can stimulate microglia, leading to neuroinflammation and worsening cognitive performance. Preliminary investigations assessing IL-18 levels, namely IL-6, demonstrated strong associations with the occurrence and intensity of HE [168]. An IL-6 level above 11 pg/mL is a potentially effective threshold for identifying MHE [49]. Other studies have also examined the role of S100β as a biomarker in HE, specifically in the context of acute liver failure (ALF) [169]. During ALF, the concentration of S100β protein in the blood increased by up to three times upon admission and remained elevated for 72 hours [170]. However, there was no association between S100β levels and the severity of HE, the occurrence of brain herniation, or the final result [171]. Two smaller studies in patients with CLD found that individuals without HE had normal S100β levels. However, patients with HE showed a twofold increase in S100β levels. S100β levels above 0.13 µg/L achieve 83% sensitivity and 64% specificity for detecting HE. The correlation between S100β and HE severity was not seen, indicating its inability to differentiate between OHE and MHE [172,173].

Evaluation for Precipitating Causes

Numerous studies have documented multiple risk factors, which can be categorized into two main groups: precipitating events and predisposing factors (Table 2).

**Table 2:** Precipitating factors diagnosis and management BRTO: Balloon-Occluded Transvenous Retrograde TIPS: transjugular intrahepatic portosystemic shunt, SPSS: spontaneous portosystemic shunt, BRTO: balloon-occluded retrograde transvenous obliteration, CARTO: coil-assisted retrograde transvenous obliteration, PARTO: plug-assisted retrograde transvenous obliteration, HE: hepatic encephalopathy, IV: intravenous, BMI: body mass index, DEXA: dual-energy X-ray absorptiometry, GI: gastrointestinal, PPI: proton pump inhibitor.Obliteration, CARTO: Coil-Assisted Retrograde Transvenous Obliteration, PARTO: Plug-assisted BRTO.

Precipitant factor	Diagnosis	Management
Alcohol and drugs	Anamnesis	Alcohol consumption counseling and discontinuation of hepatotoxic drugs
Constipation	More than 24 hours without passing stool or demonstration of significant fecal retention in the colon	Oral laxatives and/or cathartics, bowel enemas
Dehydration	Any sign of dehydration (skin and mucosal dryness, confused state) in a suitable context (patient with vomiting, diarrhea, diuretics abuse), as well as sodium, creatinine, and hematocrit increase	Correct dehydration (fluid therapy, stop diuretics)
Portosystemic shunt (spontaneous and TIPS)	Evidence of SPSS at radiologic imaging. Anamnesis and/or evidence of TIPS at radiologic imaging	Radiological shunt obliteration (BRTO, CARTO, PARTO) in case of recurrent/persistent HE
Electrolyte disorder	Hyponatremia (sodium < 130 mEq/L)	Hypovolemic hyponatremia: administration of normal saline. Hypervolemic hyponatremia: fluid restriction (<1000 mL/day); consider hypertonic saline or albumin administration.
	Hypokalemia (potassium < 3 mEq/L)	Administer oral or IV potassium supplementation
Malnutrition	Non-instrumental tools: BMI, food diary, objective examination, biochemical parameters, anthropometric measurements, creatinine-to-weight ratio, subjective global assessment questionnaire. Instrumental tools: bioimpedance test, handgrip strength test, DEXA, computed tomography scan	Dietary consult and advice for a correct supply of nutrients. Physical exercise to improve muscle tropism based on the patient’s potential
Gastrointestinal bleeding	Any evidence of upper GI tract bleeding (hematemesis or melena)	Variceal bleeding: stabilize the patient if unstable, start vasoactive drugs and antibiotic prophylaxis, and perform an upper endoscopy. Consider TIPS placement in selected cases. Non-Variceal Bleeding: stabilize the patient if unstable, start a PPI IV at a high dose, perform upper endoscopy within 24 h (<12 h in high-risk patients), and perform endoscopic hemostasis if indicated.

Age, liver function, the existence of portal hypertension, medication, renal function, genetic predispositions, underlying medical conditions, and the pathophysiology of liver cirrhosis (alcoholic or nonalcoholic) are risk factors [12]. Common risk factors that might trigger or worsen the condition include problems associated with decompensated liver cirrhosis, such as acute-on-chronic liver failure, hepatorenal syndrome, and gastrointestinal hemorrhage [174]. Additional risk factors involve infection, hypokalemia, hyponatremia, constipation, disruption of the gut-liver axis, type 2 diabetes mellitus, malnutrition, and sarcopenia [88,175]. Certain pharmacological substances can worsen HE, such as opioids, proton pump inhibitors, benzodiazepines, and other sedative medications. Infection is the primary precipitating factor, while electrolyte imbalance is the secondary precipitating factor [176]. All patients diagnosed with HE who were admitted to the intensive care unit (ICU) had a minimum PF, and the majority had multiple PFs. Increased mortality was independently associated with PF or an infection [177].

Moreover, there is a direct relationship between the severity of portosystemic shunt and an increased likelihood of HE. A study discovered that individuals with cirrhosis with a total cross-sectional spontaneous portosystemic shunting area greater than 83 mm2 had a higher risk of experiencing HE [178]. In individuals with cirrhosis, some genetic risk factors, including single-nucleotide polymorphisms in fucosyltransferase 2, toll-like receptor 9, solute carrier family 1 member 3, and solute carrier family 1 member 5, have been linked to an increased likelihood of developing HE. Moreover, these genetic risk variables have demonstrated the ability to estimate the intensity of HE [179]. Furthermore, patients carrying genetic variations in the promoter region of GLS1, which encodes kidney-type glutaminase, had a higher occurrence of HE [180]. This finding establishes a connection between specific genes and an elevated HE risk in patients who have experienced previous episodes [12].

Challenges in Diagnosing MHE

In fact, there needs to be more comprehensive knowledge on how to combine different test methodologies and interpret the results effectively. Additionally, studies have often needed better agreement between different tests. Diagnosing HE poses challenges. Clinical scales have been criticized for their poor specificity and significant variability among operators, particularly for mild HE [181]. MHE remains a condition that is not diagnosed frequently enough. This might be attributed to the limited quantity of multicentre randomized studies, the need for definitive guidelines for suggested diagnostic methods, and a shortage of experienced professionals [182]. Variation is wide due to the absence of standardized research methodologies and the unique characteristics of the examined populations. Although patients with MHE may not exhibit clear clinical symptoms, it still significantly impacts their everyday functioning. At the same time, Individuals diagnosed with MHE experience a notable decline in their overall well-being and quality of life as it relates to their health [69]. The diagnosis of MHE can be ignored due to the predominant impairments in global cognitive functioning and motor skills, while linguistic abilities typically remain intact. A survey conducted among members of the American Association for the Study of the Liver revealed that while 84% of participants recognized the clinical significance of MHE, nearly 40% of respondents had never undergone testing for MHE [183].

Management

Pharmacological Management

The recommendation is that only OHE should be routinely treated [17]. The target of treating the first attack of OHE should be limiting the result, reducing the duration, and treating the cause (Table 3). After that, we should focus on limiting hospital stays, preventing recurrence, and improving quality of life [184,185]. Treatment targets decreasing ammonia production and stimulating its elimination [186]. However, preventing recurrence by focusing on the related factors should also be assessed.

**Table 3:** Published studies on the management of minimal hepatic encephalopathy BCAA: branched-chain amino acids, BDT: block design test, CFF: critical flicker frequency, CEP: cognitive evoked potentials, DST: digit symbol test, EEG: electroencephalogram, FOS: fructooligosaccharide, HRQOL: health-related quality of life, ICT: inhibitory control test, LTT: line tracing test, MHE: minimal hepatic encephalopathy, NCT-A: number connection test-A, NCT-B: number connection test-B, NfL: Neurofilament light, OHE: overt hepatic encephalopathy, PHES: psychometric hepatic encephalopathy score, SDT: serial dotting test, SIBO: small intestinal bacterial overgrowth.

Reference	Population	Intervention	Comparison	Outcomes
Lactulose therapy				
Morgan et al. (1989) [187]	14/14	Lactulose	Lactitol	No differences between treatments in the median change in psychometric time or scores
Horsmans et al. (1997) [188]	7/7	Lactulose	Lactose as placebo	Improvement in time on the psychometric test on lactulose group with respect to basal values. Rate of improvement NCT: 5/7 (71.42%) vs 1/7 (14.28%); NNT:2
Watanabe et al. (1997) [189]	22/36	Lactulose	No treatment	MHE had disappeared in 10 (50%) of the 20 treated patients at week 8, but it persisted in 11 (85%) of the untreated 13 patients
Dhiman et al. (2000) [190]	14/12	Lactulose	No lactulose	Improvement in 8/10 (80.0%) vs 0/8 (0.0%), P < 0.001)
Prasad et al. (2007) [191]	31/30	Lactulose	No treatment	ITT analysis: Improvement in 20/31 (64.5%) vs 2/30 (6.7%); NNT:2
Bajaj et al. (2012) [192]	1000	Lactulose	Rifaximin	Suggestions: MHE diagnosis with ICT and treatment with lactulose
Sharma et al. (2012) [193]	105/55	Lactulose	No lactulose	Improvement of MHE in 21/32 (66%) vs. 9/36 (25%)
Ziada et al. (2013) [194]	24/26	Lactulose or Probiotic	No treatment	Normalization of psychometry in half of treated patients (13/24 vs. 14/26 vs. 3/25). Reduction of OHE risk development
Pratap et al. (2015) [195]	40/33	Lactulose or probiotic	No treatment	Psychometric improvement in 23/33 and in 25/40. Improvement in MHE correlated with a reduction in ammonia levels.
Sidhu et al. (2016) [196]	55/57	Lactulose	Rifaximin	MHE reversal in 38/55 and in 42/57; HRQoL was significantly improved in both groups
Wang et al. (2019) [197]	67/31	Lactulose	No lactulose	ITT analysis: 43/67 (64.2%) vs 7/31 (22.6%); NNT: 3PPS: 41/59 (69.5%) vs 6/28 (21.4%); NNT: 2
Wang et al. (2023) [198]	52 (Pro, n = 34) (Rif, n = 11) (Lac, n = 7)	Probiotics, Rifaximin, Lactulose	Baseline before Probiotics, Rifaximin, Lactulose	MHE Recovery Rates: Probiotics: 58.8% (20/34); Rifaximin: 45.5% (5/11); Lactulose: 57.1% (4/7); No significant difference in efficacy between the three groups (p = 0.843).
Rifaximin therapy				
Sidhu et al. (2011) [199]	49/45	Rifaximin	Placebo	Reversal at 2 wk: 57% vs 18% (NNT: 3); At 8 wk: Reversal of 75.5% vs 20% (NNT: 2)
Ballester et al. (2022) [200]	32	Rifaximin	Patients without metabolic syndrome manifestations	Mean PHES in responders (21 patients, 66%) was −7.3 ± 0.7, −3.2 ± 0.3, and −3.3 ± 0.6 at study initiation and at 3 and 6 months of rifaximin, respectively
Bajaj et al. (2013) [201]	20	Rifaximin	Baseline (before Rifaximin)	Significant improvement in cognitive performance (6/7 psychometric tests)
Ahluwalia et al. (2014) [202]	20	Rifaximin	Baseline (before Rifaximin)	Changes in ICT, improvement of 12% respect to baseline, indicating a better cognition
Zhang et al. (2015) [203]	26	Rifaximin	Baseline (before Rifaximin)	MHE reversal in 15/26; reduction in SIBO and ammonia levels
Goyal et al. (2017) [204]	42/38	Rifaximin	Lactulose	Still free of MHE: Rifaximin 42.8% vs lactulose 50.0% (NNT: 14)
Bakulin et al. (2023) [205]	258	Rifaximin	Cyclic therapy	Continuous rifaximin-alfa therapy significantly improves cognitive function and quality of life in patients with liver cirrhosis and MHE compared to cyclic therapy
Fiorillo et al. (2023) [206]	31	Rifaximin	Patients without MHE, controls, baseline (before Rifaximin)	NFL levels in MHE patients who responded to the treatment significantly decreased from 16.1 ± 2.7 to 11.9 ± 1.8
Casanova-Ferrer et al. (2024) [207]	18/57	Rifaximin	No treatment	Rifaximin treatment improved cognitive functions, as evidenced by better scores on the PPHES, OSDMT, and Stroop tests.
L-Ornithine L-Aspartate (LOLA)				
Kircheis et al. (1997) [20]	26/27	LOLA infusion	Placebo	Improvement in the mean time to respond NCT-A from baseline (29% vs. 9.73%)
Branched-chain amino acid (BCAA) therapy				
Egberts et al. (1985) [208]	11/11	BCAA	Placebo	Improvement in the psychometric test from 0 to 13.63% with respect to basal values in DST
Plauth et al. (1993) [209]	17	BCAA	Placebo	BCAA is superior to placebo in Psychometry and driving ability
Marchesini et al. (2003) [210]	174 patients with advanced cirrhosis	12-month BCAA supplementation	Lactoalbumin or maltodextrins	BCAA significantly reduced the combined event rate (death or deterioration) compared to lactoalbumin. The BCAA group had fewer hospital admissions and improved nutritional parameters, liver function, anorexia, and quality of life.
Les et al. (2011) [211]	116 patients with cirrhosis and prior HE episode	56-week supplementation with 30 g BCAA	Maltodextrin	There was no significant difference in HE-free survival. The BCAA group showed better performance on two neuropsychological tests and increased mid-arm muscle circumference.
Probiotic therapy				
Liu et al. (2004) [32]	40 patients with cirrhosis and MHE. 15 healthy volunteers (controls).	Probiotic, Prebiotic	Baseline (before synbiotic administration) Healthy controls	MHE Reversal: Synbiotic group: 50% (20/40) showed significant improvement in psychometric test scores. No significant changes in the placebo group. Blood Ammonia: Significant reduction in arterial and venous ammonia levels in the synbiotic group (p < 0.01). No significant change in the placebo group. Endotoxemia: Significant reduction in plasma endotoxin levels in the synbiotic group (p < 0.01). No significant change in the placebo group.
Malaguarnera et al. (2007) [212]	30/30	Bifidobacterium longum + FOS	Placebo	No statistical or clinical change was found with respect to basal values at 30, 60, 90, and 120 days
Bajaj et al. (2008) [213]	17/8	Probiotic yogurt	No treatment	ITT analysis: Reversal in 12/17 (70.58%) vs 0/7 (0%)
Sharma et al. (2008) [214]	31/31	Lactulose or probiotic or lactulose + probiotic	No treatment	Normalization of all parameters in half of the treated patients (17/31, 16/31, and 17/30, respectively)
Mittal et al. (2011) [215]	40/40/40/40	Lactulose/VSL#3, LOLA	No treatment	ITT analysis: Reversal of 4 (10%) in the no treatment group, 19 (47.5%), 14 (35%), and 14 (35%)
Bajaj et al. (2014) [216]	14	Probiotics	Placebo	Reduction in endotoxin and TNF-α but not in cytokines. No effects on psychometric performance
Lunia et al. (2014) [217]	86/74	Probiotic	No treatment	Probiotics prevented HE. Reduced arterial ammonia, SIBO, OCTT; increased PHES and CFF thresholds compared to control.
Sharma et al. (2014) [218]	32/31/31/30	Probiotics	LOLA, rifaximin, placebo	All three treatments were better than placebo in improving CFF and abnormal neuropsychiatric test results. Rifaximin and LOLA significantly improved CFF compared to placebo.
Pratap Mouli et al. (2015) [195]	33/40	Probiotic	Lactulose	Probiotics non-inferior to lactulose in improving MHE. Improvement in both groups correlated with a reduction in ammonia levels.
Xia et al. (2018) [219]	30/37	Probiotic	No treatment	Improved cognition, enriched Clostridium cluster I and Bifidobacterium, decreased Enterococcus and Enterobacteriaceae. Reduced venous ammonia and improved intestinal mucosal barrier parameters.
Combined therapy				
Liu et al. (2004) [32]	20/20/15	Symbiotics + fermentable fibers	Fermentable fibers/placebo	Reversal of 50% in the symbiotic group, 50% in the fermentable fibers group, and 13% in the placebo. Not statistically significant until compression of treatment groups vs placebo (P = 0.03)
Ndraha et al. (2011) [220]	17/17	BCAAs + LOLA	BCAAs	Improvement in CFF 7.0% and 1.96% values (Hz), with respect to baseline
Kato et al. (2013) [221]	19	30-35 Kcal + 1.0-1.5 g/kg of protein/d	baseline (before treatment)	11/19 (57.8%) recovered at 4 wk, and 13/19 (68.4%) at 8 wk
Rai et al. (2015) [222]	7	Lactulose and Rifaximin	Controls, NMHE group, baseline (before treatment)	Psychometric improvement (P=0.044); reversal of low-grade cerebral edema
Shi et al. (2023) [223]	44/44	A combination of probiotics and lactulose.	Lactulose alone.	A combination of probiotics and lactulose is more effective than lactulose alone in improving cognitive function and quality of life in patients with MHE
Others				
Malaguarnera et al. (2008) [224]	60/55	Acetyl-L-carnitine	Placebo	Changes of mean values in at least 20.71% to 32.79% with respect to basal values
Agrawal et al. (2011) [225]	35	For patients with MHE and H. pylori infection, Clarithromycin, lansoprazole, and tinidazole	Relationship between H. pylori and MHE, rather than directly contrasting different treatment approaches.	Improvement in 12.7%, 13.3%, and 18.7% with respect to basal mean time in NCT, FCT, and LTT
Bajaj et al. (2014) [216]	18/19	Lactobacillus GG	Placebo	Improvement from 1.02% to 15.89% from baseline values
Burkard et al. (2013) [226]	25	Potassium–iron–phosphate–citrate	baseline (before treatment)	Normalization of psychometry in 72% vs. 26.9%. QoL improvement.
Maharshi et al. (2016) [114]	60/60	Nutritional education	No nutritional therapy	27/38 (71.1%) vs 8/35 (22.8%); 27/60 (45%) vs 8/60 (13.3%) when considering PPS and ITT analysis.
Janyajirawong et al. (2021) [227]	34/32	Zinc supplementation	Placebo	A 12-week course of zinc supplementation significantly improved psychomotor performance in cirrhotic patients with MHE. Patients receiving zinc performed better in neuropsychiatric tests (NCT-A, NCT-B, SDT, DST) than placebo patients.
Sharma et al. (2023) [228]	75/75	Nutritional therapy	No nutritional therapy	Nutritional therapy is effective in treating MHE and is associated with improving nutritional status, HRQOL, ammonia, endotoxins, inflammatory markers, and myostatin levels.

Nondigestible lactitol, lactulose, and disaccharides are the first line of treatment. These substances are broken down into organic acids (of short chain) in the colon [5]. Acidity reduces the bacteria that produce ammonia, thereby decreasing ammonia production and converting ammonia into ammonium (nonabsorbable), thereby decreasing its absorption. Also, they increase osmolarity, leading to rapid intestinal transit, and remove excess nitrogen from feces through their laxative effect [184-186]. Lactose additionally increases zinc absorption [229]. Lactulose enema improves electroencephalograms and asterixis to the same degree as neomycin tablets, but lactulose enema also acidifies stool pH, thus improving the number connection test [230]. It was proven that there was nearly no difference between lactulose and lactitol regarding efficacy and safety [231]. Gluud et al. found that nonabsorbable disaccharides play a role in HE treatment and mortality [232]. PEG or polyethylene glycol 3350-electrolyte solution is an osmotic laxative that can be used instead of lactulose in the treatment of hospitalized HE patients [233,234].

Antibiotics targeting the reduction of the number of intestinal bacteria that produce ammonia can be used in therapy [186]. The first antibiotic to be used was neomycin, which reduced the concentration of colonic ammonia [235]. Rifaximin (non-absorbable antibiotic) inhibits RNA synthesis in gram-negative, gram-positive, anaerobic, and aerobic intestinal bacteria [184]. Rifaximin was proven to be as effective as neomycin and also as effective and safe as lactulose [236,237]. Also, studies proved that the use of rifaximin plus lactulose has a better response than lactulose alone in the treatment of HE and mortality reduction [238]. Rifaximin was found to have the same effect as nonabsorbable disaccharides or other antibiotics but with more safety in asterixis reduction and improving the mental state [239].

Nutritional Therapies and Dietary Modifications

Metabolic ammonia scavengers are substances that increase the excretion of ammonia in urine through an alternative pathway. One of the firstly used scavengers was sodium benzoate, which is conjugated in the liver with glycerin and then passed in urine as Hippurate [240]. It has proved to be as effective as nonabsorbable disaccharides in treating HE [241]. Glycerol phenylbutyrate also eliminates ammonia through excretion in the urine as phenylacetylglutamine. Ammonia levels with glycerol phenylbutyrate were lower than with rifaximin and lactulose [238]. Ornithine phenylacetate activates glutamine synthetase, inducing muscle trap of ammonia (as glutamine) followed by its conjugation with phenylacetic acid, forming phenylacetylglutamine and then excreted in urine [242]. Ornithine phenylacetate administration after GIT bleeding (upper tract) reduces ammonia levels in the plasma and appears to be safe [243].

LOLA converts ammonia into glutamine and urea, thus reducing the level of blood ammonia [185,186]. Oral LOLA appears to have the same effect as lactulose in improving asterixis, mental state, and reducing ammonia levels [244]. A Cochrane review found that LOLA reduces mortality and improves HE, but there is still no clear recommendation for its use [245].

The liver synthesizes albumin protein, which modulates the anti-inflammatory response and detoxification [5,184]. Studies have shown that combining albumin with lactulose is more effective than lactulose alone for treating HE and reducing mortality [246].

Probiotics are living microorganisms that, at a certain level, have a beneficial role in the host through modulating GIT immunity [247]. Some probiotics can reduce inflammation, restore the microbiota, and prevent bacterial translocation by maintaining the integrity of the intestinal barrier [248]. VSL#3 was the most commonly used probiotic [249]. The use of fibers with probiotics appeared to reverse the minimal HE [250]. Studies have shown that combining lactulose with probiotics can reduce the risk of unimprovement in HE patients [251].

The nutritional state, portosystemic shunt, and level of decompensation are very important and affect the patient's response to diet. Studies found no difference in the effects of vegetable protein (*Plantago psyllium*) compared with animal protein in patients with type 2 DM and HE [252]. The current recommendations suggest an intake of 35-40 kcal/kg of body weight and 1.2-1.5 g/kg of protein per day, regardless of the presence or absence of HE, and recommend consuming both vegetable and animal protein [253].

Role of Liver Transplant

Persistent HE, especially when liver function is not severely affected, suggests portocaval shunts (spontaneous). These patients can benefit from embolization, as evidenced by a decrease in the number of HE attacks [254]. Also, shunt embolization was associated with better survival in these patients if MELD score < 15 and did not have hepatocellular carcinoma [255]. Liver transplantation may be a treatment option in some HE patients [184].

Microbiota plays an important role in the pathogenesis of HE. A trial showed that patients using standard lines of therapy had a poorer response than those using transplanted fecal microbiota. Also, cognition was improved compared to the standard [256]. The neurological state also improved, and the hospitalization period decreased with it [33]. However, there is still no recommendation for its use [257].

Low zinc levels can increase the ammonia level in the blood by impairing enzyme activity in the urea cycle and glutamine synthetase. Zinc supplements were associated with improvement in the number connection test but did not affect HE recurrence [258].

Lactulose plus rifaximin is considered the best prophylaxis against HE recurrence [259]. Studies have also proved that lactulose is a very strong prophylactic agent for preventing HE after variceal bleeding (acute attack)[260]. Also, using rifampicin can decrease the hospitalization rate in the long run [261]. Long-term use of albumin can also prevent HE recurrence and reduce the incidence of grades 3 and 4 of HE [262].

Patients with end-stage liver failure may suffer from HE and confusion. This is due to manganese accumulation in the pituitary gland, mesencephalon, and lentiform nucleus, which can be seen using MRI. After liver transplantation, these changes disappear due to the functioning of a new liver [263]. Some studies showed that the degree of HE before liver transplantation affects the degree of awareness after transplantation [264-266]. After transplantation, cases with fourth-stage HE need nearly five days for recovery [266,267]. Intraoperative brain ischemia, permanent Ischemia to the brain with or without persistent shunt before transplantation, can make HE irreversible after transplantation [268-270]. Studies proved that after liver transplantation, HE will show incomplete regression. So, it is necessary to develop different treatment plans after transplantation for complete HE recovery [271].

Interesting points of discussion

Prevalence and Incidence of MHE

MHE, which is the mildest subtype of HE, is associated with psychomotor and cognitive affection with no clinical symptoms of HE [272]. Zeegen et al. first described MHE, in which it was found that many patients who underwent surgery for portal decompression had abnormal scores (in trail-making tests) [273]. After nearly eight years, the definition of subclinical HE appeared in the literature. The definition of OHE appeared to combine the first grade of HE with MHE [274].

The incidence of MHE ranges between 20 to 80 % among cirrhotic patients early or during the disease course. This wide range is due to different tests and different diagnostic symptoms [191,275]. The prevalence of HE is about 10 to 14 % at the time of cirrhosis diagnosis and about 16 to 21% in patients with decompensated cirrhosis. It reaches about 50% after an intrahepatic portosystemic shunt. The risk for the first attack of HE within five years of cirrhosis diagnosis can reach about 25% [184].

Impact of MHE on Quality of Life

Liver disease affects the patient's health and quality of life. The most affected activities are psychomotor skills, information processing, and those requiring attention. However, usual activities such as dressing, shopping, and maintaining personal hygiene are maintained [191]. Several mechanisms may impair the quality of life among cirrhotic patients. Impairment of physical performance forces these patients to limit their activities, especially in the late stages of the disease. Also, cirrhosis complications such as bleeding and ascites can impair quality of life. Marchesini et al. showed that cirrhosis symptoms such as muscle cramps and itching can strongly affect quality of life [276]. Disease severity, sleep disorders, and hospitalization can also affect the quality of life [277,278]. Also, Nardelli et al. showed that anxiety, alexithymia, and depression were common among cirrhotic subjects and widely affect HRQOL [279]. MHE patients had a reduction in psychological sub-score, total score, physical subscore, and all 12 SIP scales. Groenweg et al. reported that compared to those who do not have MHE. An analysis showed that MHE was associated with a low SIP scale in the areas of social interaction, work, recreation, and vigilance [67]. Schomerus et al. reported that about half of MHE patients could not work [280]. Also, loss of appetite and MHE among cirrhotic patients can negatively affect HRQOL [69].

The question now is whether a specific therapy for MHE can improve HRQOL [67,276,281]. The prevalence of sleep disorders among cirrhotic patients varies from 26% to 70%, which greatly affects the health and quality of life [72,282]. Also, sleep disorders can badly affect the liver function and cognitive level [283]. Melatonin clearance is decreased due to the affected liver (hepatic clearance), so the level increases daily. Also, defects in the circadian rhythm in melatonin with a delayed nocturnal peak in plasma can likely occur due to decreased sensitivity to light [71]. However, other mechanisms, such as thermoregulatory and neuromuscular alteration, may contribute to this [284].

Cirrhotic patients have a very high risk for falls [285]. The possible causes may be sleep problems, changes in body composition such as myosteatosis and sarcopenia, malnutrition, antidepressant medications, cognitive dysfunction, and endocrine disorders such as hypogonadism. These fractures usually lead to hospitalizations, fractures, high costs for health care, and thus bad HRQOL. The result is more serious among cirrhotic patients due to coagulopathy and the risk of operation [79].

Urios et al. reported that MHE patients have a higher risk of falls due to delayed movement onset, increased reaction time, impaired postural control, and decreased stability [286]. All these factors could contribute to bradykinesia among MHE patients [287]. Also, cirrhotic patients have very bad outcomes after accidents (mainly traffic accidents) with long hospital stays, high costs for health care, and higher mortality rates [62]. Furthermore, the decreased ability to work among these patients puts an economic burden on them and their families, which may increase the indirect cost of this disease [77].

Continuum of HE From MHE to OHE and Prognosis

MHE is a risk factor for OHE and also for mortality [288]. Within nearly three years, about 50% of patients suffer from OHE [275]. Hartmann et al. reported an increased risk of OHE among MHE patients after nearly 29 months and more attacks of OHE than those not having cognitive defects, with no difference in mortality risk between the two groups [289]. Also, other studies proved that patients with MHE are at higher risk for developing OHE, especially those having high levels of ammonia and higher severity of the disease [163]. The incidence of OHE is higher among MHE patients with impaired function of the liver and those in the late stages of cirrhosis [275]. PHES of less than six and Child-Pugh score above seven were connected with higher mortality, as Dhiman et al. reported [89]. In multivariate analysis, Amodio et al. revealed that MHE had a prognostic effect on survival and disease severity [148].

MHE Versus HE Stage I in Clinical Practice

HE is classified clinically into groups according to the West Haven criteria, which considers neuromuscular problems, changes in personality, and alteration in both intellectual function and consciousness [136]. Unfortunately, the diagnosis of grade 1 HE clinically according to these criteria is suggested to be operator-dependent; from this point, the expression of OHE appeared when at least flapping tremors or temporal disorientation occurred [136,290]. This flapping tremor is a negative myoclonus, not a true tremor. When grade 1 HE, in which the patient is symptomatic, is grouped with MHE, in which the patient has only abnormal tests without clinical symptoms, the term Covert HE arises. However, this term made a huge debate as covert means hidden, although these patients are clinically symptomatic [181]. It was also noted that this condition has a different prognosis than MHE [291]. MHE diagnosis is very important as it is a very common condition and is associated with an increased risk of OHE and a worse prognosis [163,280]. In general, patients with both grade 1 HE and MHE are likely to have the worst prognosis among cirrhotic patients with no neuropsychiatric symptoms [292].

Future perspectives and current trends

Development of Biomarkers

Identifying reliable biomarkers for MHE can significantly improve early diagnosis, intervention, and patient management. A study examined the roles of S-100β and NSE in MHE patients undergoing LOLA treatment. However, these biomarkers did not significantly correlate with MHE severity [293]. Labenz et al. showed that higher serum levels of neurofilament light chains were independently associated with the presence of MHE, demonstrating reliable discriminative power for MHE detection [294].

Another promising biomarker for MHE is 3-nitrotyrosine, which has high sensitivity and specificity for detecting MHE in cirrhotic patients [295]. Also, brain-derived neurotrophic factor (BDNF) serum levels were twofold lower in patients with liver cirrhosis than in healthy subjects, a decrease attributed to a degree of liver insufficiency that may serve as a potential diagnostic marker for MHE [296].

The progress of artificial intelligence in the field of medical imaging offers the opportunity to discover more efficient diagnostic biomarkers and enhance the predictive accuracy of MHE and, with a significant role of modern MRI techniques, have the potential to offer new perspectives for the future detection of MHE [297]. In addition, Luo et al. suggested that the intestinal microbiota compositions of MHE patients were shown to be changed, specifically in terms of the relative abundances of Salivarius and Veillonella. These changes were linked to sleep disorders, indicating that Salivarius and Veillonella could be used as diagnostic biomarkers [298]. Gairing et al. suggest that IL-6 could be useful for identifying individuals who should undergo further psychometric testing, thereby eliminating the necessity for such testing in around one-third of cases. Furthermore, when IL-6 serum levels reach or exceed seven pg/mL, it may be appropriate to consider starting anti-HE medication in patients unable to undertake psychometric testing [299]. Collectively, a variety of biomarkers are present, but the actual challenge is to ensure accuracy and reliability using larger population studies and making more effort to identify new biomarkers.

Development of Medical Calculators

Dynamic functional connectivity strength (DFC-Dstrength) is a resting-state functional magnetic resonance imaging (rs-fMRI)-derived metric that quantifies the strength of time-varying functional connectivity between different brain regions using a sliding-window approach. Unlike conventional static functional connectivity analyses, DFC-Dstrength captures the dynamic fluctuations in neural network interactions over time, thereby providing greater sensitivity to subtle alterations in brain function. In the study by Cheng et al., DFC-Dstrength demonstrated the highest classification accuracy among the evaluated dynamic connectivity metrics for distinguishing patients with MHE from cirrhotic patients without HE (noHE). Using the DFC-Dstrength metric, seven nodes were identified as the most discriminative features for classifying MHE from noHE, including the left inferior parietal lobule, left supramarginal gyrus, left calcarine, left superior frontal gyrus, left cerebellum, right postcentral gyrus, and right insula. Overall, DFC-Dstrength-based features exhibited greater diagnostic precision than conventional static connectivity measures in identifying MHE in patients with cirrhosis [300].

Another study suggests that cirrhotic individuals underwent eye testing with the OSCANN desk 100. The main features were derived from the memory-guided saccades, smooth pursuit, and antisaccades tests. An algorithm achieved a sensitivity of 93% and a specificity of 93%, exceeding the performance of the current gold standard, the PHES battery test [301].

Preventive Medicine

Based on the concept that HE refers to a range of cognitive and behavioral impairments that can occur as a complication of cirrhosis and have negative effects on outcomes. Ammonia is essential for its development. Rifaximin is a non-absorbable antibiotic that prevents the growth of bacteria that produce urease and decreases the intake of ammonia from food and bacteria. Rifaximin has the potential to both prevent and treat HE in individuals with cirrhosis. However, its efficacy is dependent on the specific state of the patient [302]. Furthermore, a combination of rifaximin and lactulose may be beneficial in preventing HE after TIPS placement, and most medications are ineffective alone [303].

Modern diagnostic tools such as PHES, CFF, and ANT are used to detect and grade MHE at the initial stages. This can help intervene in time and prevent it from progressing to the OHE stage. However, PHES is not usually used in clinical practice due to its time-consuming nature. Hence, it is necessary to create efficient diagnostic algorithms to advance screening and therapy [105]. In addition, Probiotics were more effective than placebo or no treatment in reversing MHE and reducing serum ammonia levels in patients with MHE. Still, they were less effective than lactulose or LOLA, underscoring the need to highlight their role in preventive treatment strategies [304]. Utilizing comprehensive strategies, such as the ideal transitions of care model, can effectively reduce the risk of readmission among patients with MHE, facilitating a smooth transition to outpatient care. Ongoing research is focused on developing innovative approaches to reduce readmissions among patients with HE and to enhance their quality of life [305]. Expanding upon the integrated approach in the optimal transitions of care framework, advancements in digital and technology can result in additional enhancements in healthcare delivery. An example of such a method is the utilization of electronic medical record (EMR) alerts and electronic checklists. A quality improvement study examined the effectiveness of a paper checklist vs an electronic checklist in managing different decompensations of cirrhosis, with a specific focus on HE [12].

## Conclusions

MHE is a major challenge in the management of CLD because it affects a large number of patients and often remains unrecognized because of its subtle clinical presentation. The present review sheds light on some of the interrelationships among factors involved in the pathogenesis of MHE, from the central role of hyperammonemia and its neurotoxic effects to complex systemic inflammation, gut dysbiosis, and altered neurotransmission. While no single diagnostic test perfectly captures the multifaceted nature of MHE, combining psychometric assessments, such as the PHES, with neurophysiological tests, such as EEG and critical flicker frequency (CFF), as outlined in this review, contributes to accurate detection. Strategies for reducing the ammonia burden and managing MHE include nonabsorbable disaccharides, such as lactulose, and antibiotics, such as rifaximin. These are usually combined with nutritional therapies and probiotics. In selected cases, treatment may include embolization of portosystemic shunts or even liver transplantation. Looking ahead, the development of dependable biomarkers, AI-driven diagnostic algorithms, and preventive strategies, including gut microbiome modulation, may enable the early diagnosis of MHE and improve the quality of life and long-term outcomes of patients with CLD.
